# Supplementary material for: Variation in guide RNA library representation results in gene effect score bias in genome-wide CRISPR screens
Source: BMC Genomics. 2026 Feb 20;27:307. doi: 10.1186/s12864-026-12658-2 (PMC13023172; doi:10.1186/s12864-026-12658-2)
Supplement: Supplementary file 3 — Supplementary Material 3 [file 12864_2026_12658_MOESM3_ESM.pdf]

Variation in guide RNA library representation results in gene effect score bias in genome-wide CRISPR screens.

Paul Metz[1∫], Sofia Alves-Vasconcelos[1], Richard Wallbank[1], Joey Riepsaame[1,2], Stuart Brown[1] and A. Bassim Hassan[1,¶].

[1]Oxford Molecular Pathology Institute, Sir William Dunn School of Pathology, University of Oxford, South Parks Road, Oxford, OX1 3RE, United Kingdom.
[2]Genome Engineering Oxford, Sir William Dunn School of Pathology, University of Oxford, South Parks Road, Oxford, OX1 3RE, United Kingdom.

Supplementary Tables

Supplementary Table 1. GSEA of combined transcriptomics of STS26T, 90-8, ST-8814 and iHSC cells cultured in HPLM versus DMEM.

Gene set	Enriched in	FDR q value (<25%)
Hallmark_cholesterol_homeostasis	DMEM	0.024
Hallmark_peroxisome	DMEM	0.072
Hallmark_wnt_beta_catenin_signaling	DMEM	0.245
Hallmark_androgen_response	DMEM	0.214
Hallmark_adipogenesis	DMEM	0.194
Hallmark_mtorc1_signaling	DMEM	0.191
Kegg_ribosome	HPLM	0.14
Kegg_protein_export	HPLM	0.225
Kegg_hematopoietic_cell_lineage	HPLM	0.179
Kegg_type_i_diabetes_mellitus	HPLM	0.181
Kegg_parkinsons_disease	HPLM	0.155
Kegg_oxidative_phosphorylation	HPLM	0.155
Kegg_biosynthesis_of_unsaturated_fatty_acids	DMEM	0.055
Kegg_ppar_signaling_pathway	DMEM	0.078
Kegg_endometrial_cancer	DMEM	0.136
Kegg_inositol_phosphate_metabolism	DMEM	0.102
Kegg_mtor_signaling_pathway	DMEM	0.121
Kegg_citrate_cycle_tca_cycle	DMEM	0.103
Kegg_notch_signaling_pathway	DMEM	0.105
Kegg_aminoacyl_trna_biosynthesis	DMEM	0.111
Kegg_acute_myeloid_leukemia	DMEM	0.105
Kegg_steroid_biosynthesis	DMEM	0.115
Kegg_glycosaminoglycan_biosynthesis_heparan_sulfate	DMEM	0.15
Kegg_phosphatidylinositol_signaling_system	DMEM	0.141

Kegg_non_small_cell_lung_cancer	DMEM	0.131
Kegg_b_cell_receptor_signaling_pathway	DMEM	0.228
Kegg_fc_gamma_r_mediated_phagocytosis	DMEM	0.212
Kegg_lysine_degradation	DMEM	0.209
Kegg_vegf_signaling_pathway	DMEM	0.208
Kegg_peroxisome	DMEM	0.205
Reactome_sars_cov_1_modulates_host_translation_machinery	HPLM	0.156
Reactome_srp_dependent_cotranslational_protein_targeting_to_membrane	HPLM	0.139
Reactome_eukaryotic_translation_elongation	HPLM	0.191
Reactome_activation_of_the_mrna_upon_binding_of_the_cap_binding_complex_and_eifs_and_subsequent_binding_to_43s	HPLM	0.183
Reactome_eukaryotic_translation_initiation	HPLM	0.249
Reactome_respiratory_electron_transport	HPLM	0.222
Reactome_response_of_eif2ak4_gcn2_to_amino_acid_deficiency	HPLM	0.204
Reactome_regulation_of_expression_of_slits_and_robos	HPLM	0.193
Reactome_nonsense_mediated_decay_nmd	HPLM	0.194
Reactome_complex_i_biogenesis	HPLM	0.206
Reactome_influenza_infection	HPLM	0.234
Reactome_selenoamino_acid_metabolism	HPLM	0.226
Reactome_sars_cov_1_host_interactions	HPLM	0.213
Reactome_respiratory_electron_transport_atp_synthesis_by_chemiosmotic_coupling_and_heat_production_by_uncoupling_proteins	HPLM	0.22

Supplementary Table 2. GSEA of combined CRISPR perturbation screen data from STS26T, 90-8, ST-8814 and iHSC cells cultured in HPLM versus DMEM.

Gene set	Essential in	FDR q value (<25%)
HALLMARK_UNFOLDED_PROTEIN_RESPONSE	DMEM	0.202
HALLMARK_OXIDATIVE_PHOSPHORYLATION	HPLM	0.002
HALLMARK_FATTY_ACID_METABOLISM	HPLM	0.095
KEGG_PROTEIN_EXPORT	DMEM	0.208
KEGG_PARKINSONS_DISEASE	HPLM	0.003
KEGG_OXIDATIVE_PHOSPHORYLATION	HPLM	0.001
KEGG_ALZHEIMERS_DISEASE	HPLM	0.004
KEGG_HUNTINGTONS_DISEASE	HPLM	0.004
KEGG_CITRATE_CYCLE_TCA_CYCLE	HPLM	0.059
REACTOME_TRNA_PROCESSING_IN_THE_NUCLEUS	DMEM	0.084
REACTOME_COMPLEX_I_BIOGENESIS	HPLM	0.013
REACTOME_AEROBIC_RESPIRATION_AND_RESPIRATORY_ELECTRON_TRANSPORT	HPLM	0.009
REACTOME_RESPIRATORY_ELECTRON_TRANSPORT	HPLM	0.007
REACTOME_COMPLEX_IV_ASSEMBLY	HPLM	0.195
REACTOME_ACTIVATION_OF_ANTERIOR_HOX_GENE_IN_HINDBRAIN_DEVELOPMENT_DURING_EARLY_EMBRYOGENESIS	HPLM	0.192
REACTOME_METABOLISM_OF_PORPHYRINS	HPLM	0.179
REACTOME_FORMATION_OF_ATP_BY_CHEMIOSMOTIC_COUPLING	HPLM	0.175
REACTOME_PHASE_2_PLATEAU_PHASE	HPLM	0.19
REACTOME_GLYOXYLATE_METABOLISM_AND_GLYCINE_DEGRADATION	HPLM	0.194
REACTOME_INTERLEUKIN_7_SIGNALING	HPLM	0.177
REACTOME_OXIDATIVE_STRESS_INDUCED_SENESCENCE	HPLM	0.226
REACTOME_ERBB2_REGULATES_CELL_MOTILITY	HPLM	0.233
REACTOME_RECOGNITION_AND_ASSOCIATION_OF_DNA_GLYCOSYLASE_WITH_SITE_CONTAINING_AN_AFFECTED_PURINE	HPLM	0.222
REACTOME_MEIOTIC_RECOMBINATION	HPLM	0.213
REACTOME_BASE_EXCISION_REPAIR_AP_SITE_FORMATION	HPLM	0.202
REACTOME_INHIBITION_OF_DNA_RECOMBINATION_AT_TELOMERE	HPLM	0.193
REACTOME_CHROMATIN_MODIFICATIONS_DURING_THE_MATERNAL_TO_ZYGOTIC_TRANSITION_MZT	HPLM	0.188
REACTOME_MEIOSIS	HPLM	0.178
REACTOME_FORMATION_OF_THE_BETA_CATENIN_TCF_TRANSACTIVATING_COMPLEX	HPLM	0.18
REACTOME_ASSEMBLY_OF_THE_ORC_COMPLEX_AT_THE_ORIGIN_OF_REPLICATION	HPLM	0.173

REACTOME_PRC2_METHYLATES_HISTONES_AND_DNA	HPLM	0.173
REACTOME_RUNX1_REGULATES_GENES_INVOLVED_IN_MEGAKARYOCYTE_DIFFERENTIATION_AND_PLATELET_FUNCTION	HPLM	0.167
REACTOME_DNA_METHYLATION	HPLM	0.163
REACTOME_CONDENSATION_OF_PROPHASE_CHROMOSOMES	HPLM	0.162
REACTOME_REGULATION_OF_TLR_BY_ENDOGENOUS_LIGAND	HPLM	0.171
REACTOME_BUDDING_AND_MATURATION_OF_HIV_VIRION	HPLM	0.176
REACTOME_MEIOTIC_SYNAPSIS	HPLM	0.17
REACTOME_ERCC6_CSB_AND_EHMT2_G9A_POSITIVELY_REGULATE_RRNA_EXPRESSION	HPLM	0.176
REACTOME_REGULATION_OF_ENDOGENOUS_RETROELEMENTS_BY_PIWI_INTERACTING_RNAS_PIRNAS	HPLM	0.175
REACTOME_BASE_EXCISION_REPAIR	HPLM	0.19
REACTOME_SENESCENCE_ASSOCIATED_SECRETORY_PHENOTYPE_SASP	HPLM	0.191
REACTOME_RMTS_METHYLATE_HISTONE_ARGININES	HPLM	0.187
REACTOME_REGULATION_OF_ENDOGENOUS_RETROELEMENTS_BY_THE_HUMAN_SILENCING_HUB_HUSH_COMPLEX	HPLM	0.197
REACTOME_TRANSCRIPTIONAL_REGULATION_OF_GRANULOPOIESIS	HPLM	0.21
REACTOME_FORMATION_OF_THE_NEPHRIC_DUCT	HPLM	0.227
REACTOME_DEFENSINS	HPLM	0.227
REACTOME_REGULATION_OF_INNATE_IMMUNE_RESPONSES_TO_CYTOSOLIC_DNA	HPLM	0.229
REACTOME_SURFACTANT_METABOLISM	HPLM	0.224
REACTOME_REGULATION_OF_ENDOGENOUS_RETROELEMENTS_BY_KRAB_ZFP_PROTEINS	HPLM	0.223
REACTOME_REGULATION_OF_PYRUVATE_DEHYDROGENASE_PDH_COMPLEX	HPLM	0.23
REACTOME_MATERNAL_TO_ZYGOTIC_TRANSITION_MZT	HPLM	0.235
REACTOME_DISEASES_OF_PROGRAMMED_CELL_DEATH	HPLM	0.231
REACTOME_CITRIC_ACID_CYCLE_TCA_CYCLE	HPLM	0.245
REACTOME_STIMULI_SENSING_CHANNELS	HPLM	0.248

Supplementary Table 3. CRISPR library distribution metrics: DepMap

Library	Skew Ratio	Zero-count gRNAs	Total gRNAs
Avana2	8.87	63	170,429
Avana3	12.24	50	170,429
Avana4	11.93	53	170,429
KY1	5.99	0	95,091
KY2	19.79	0	95,091
HCD	6.06	62	49,763

Supplementary Table 4. Cell lines used in this study.

Cell line	Origin, Disease	Standard medium*	Reference

STS26T	Human melanoma, sporadic	DMEM + 10% FBS + Pen/Strep	[33]
ST-8814	Human MPNST, NF1	DMEM + 10% FBS + Pen/Strep	[34]
90-8	Human MPNST, NF1	DMEM + 10% FBS + Pen/Strep	[35]
iHSC	Human, no disease	DMEM + 10% FBS + Pen/Strep	[36]
LentiX Hek293T	Human Embryonic Kidney	DMEM + 10% FBS + Pen/Strep	Takara Bio, CAT # 632180
HOS	Human osteosarcoma	RPMI + 10% FBS + 1% GlutaMax	ATCC #CRL-1543
OHS	Human osteosarcoma	RPMI + 10% FBS + 1% NEAA + 2mM GlutaMax	Obtained from Radium Hospital (NRH), University of Oslo

Supplementary Table 5. Short tandem repeat (STR) profiling of target cell lines

Marker	STS26T	90-8	ST-8814	iHSC	LentiX Hek293T
AMEL	X,X	X,X	X,Y	X,X	X,X
CSF1PO	10,13	12,12	9,12	10,11	11,12,13
D13S317	9,10	8,8	12,12	12,13	12,13,14,15
D16S539	12,13	9,9	13,13	12,12	9,14,13
D18S51	17,18	14,18	12,12	13,16	17,18
D19S433	14,14	13,16	13,14	14,14	17,18,19
D21S11	30,31	30,30	29,32.2	30.2,32.2	28,29,30.2,31.2
D2S1338	20,20	16,24	17,23	16,17	19,19
D3S1358	14,14	15,15	15,18	17,18	15,16,17
D5S818	11,12	11,11	12,13	8,11	8,9
D7S820	8,11	11,11	8,8	8,12	11,11
D8S1179	13,14	10,12	14,14	12,15	11,12,13,14,15
FGA	22,23	25,25	21,21	23,24	23,23
TH01	6, 9.3	6,6	9,9	6,7	7,9.3
TPOX	8,8	11,11	11,12	8,12	11,11
vWA	17,17	17,17	16,16	15,17	16,17,18,19,20

Supplementary Table 6. Primers used for CRISPR library validation

Primer Name	Sequence (5' to 3')
P5_ARGON_1	AATGATACGGCGACCACCGAGATCTACACTCTTTCCCTACACGACGCTCTTCCGATCTTTGTGGAAAGGACGAAACACCG
P5_ARGON_2	AATGATACGGCGACCACCGAGATCTACACTCTTTCCCTACACGACGCTCTTCCGATCTCTTGTGGAAAGGACGAAACACCG
P5_ARGON_3	AATGATACGGCGACCACCGAGATCTACACTCTTTCCCTACACGACGCTCTTCCGATCTGCTTGTGGAAAGGACGAAACACCG
P5_ARGON_4	AATGATACGGCGACCACCGAGATCTACACTCTTTCCCTACACGACGCTCTTCCGATCTAGCTTGTGGAAAGGACGAAACACCG
P7_KERMIT_1	CAAGCAGAAGACGGCATACGAGATTCGCCTTAGTGACTGGAGTTCAGACGTGTGCTCTTCCGATCTTCTACTATTCTTTCCCCTGCACTGT
P7_KERMIT_2	CAAGCAGAAGACGGCATACGAGATCTAGTACGGTGACTGGAGTTCAGACGTGTGCTCTTCCGATCTTCTACTATTCTTTCCCCTGCACTGT
P7_KERMIT_3	CAAGCAGAAGACGGCATACGAGATGCTCAGGAGTGACTGGAGTTCAGACGTGTGCTCTTCCGATCTTCTACTATTCTTTCCCCTGCACTGT
P7_KERMIT_4	CAAGCAGAAGACGGCATACGAGATAGGAGTCCGTGACTGGAGTTCAGACGTGTGCTCTTCCGATCTTCTACTATTCTTTCCCCTGCACTG
P7_MISSPIGGY_1	CAAGCAGAAGACGGCATACGAGATATTACTCGGTGACTGGAGTTCAGACGTGTGCTCTTCCGATCTACCGACTCGGTGCCACTTTTTCAA
P7_MISSPIGGY_2	CAAGCAGAAGACGGCATACGAGATTCCGGAGAGTGACTGGAGTTCAGACGTGTGCTCTTCCGATCTACCGACTCGGTGCCACTTTTTCAA

Supplementary Table 7. PCR programme used for CRISPR library validation

Stage	Temperature (°C)	Time	Number of Cycles
Initial Denaturation	98	03:00	1x
Denaturation	98	00:15	5x (Stage B)
Annealing	65	00:30	5x (Stage B)
Extension	72	00:30	5x (Stage B)
Denaturation	98	00:15	8x (Stage C)
Annealing	63	00:30	8x (Stage C)
Extension	72	00:30	8x (Stage C)
Denaturation	98	00:15	12x (Stage D)
Annealing	61	00:30	12x (Stage D)
Extension	72	00:30	12x (Stage D)
Final Extension	72	07:00	1x
Hold	4	Infinity	1x

Supplementary Table 8. Sequencing coverage, Zerocounted gRNAs, GiniIndex, and time.

	Sample ID	Reads	Mapped	Total sgRNAs	Zero count gRNAs	GiniIndex	Mean reads per construct	Cell line	Days
**1**	screen_iCH14_R1	16717078	14230507	20355	960	0.2127	699.11604	iHSC.HPLM_R1	44
**2**	screen_9DH14_R1	9132741	4321332	20355	260	0.1244	212.29831	90-8.HPLM_R1	38
**3**	screen_9DD28_R2	16666796	13783855	20355	250	0.1175	677.17293	90-8.DMEM_R2	52
**4**	screen_9CH14_R1	6727317	3631654	20355	246	0.1385	178.41582	90-8.HPLM_R1	38
**5**	screen_9CD28_R2	16926195	13631978	20355	199	0.1177	669.71152	90-8.DMEM_R2	52
**6**	screen_9CH26d_R1	17965220	1113400	20355	193	0.1165	54.69909	90-8.HPLM_R1	30
**7**	screen_8DH3_R1	20453913	13203202	20355	129	0.07411	648.64662	ST-8814.HPLM_R1	21
**8**	screen_iDH14_R1	17713794	14108677	20355	124	0.1349	693.13078	iHSC.HPLM_R1	44
**9**	screen_9CD9.5_R2	8749743	4164001	20355	119	0.1015	204.56895	90-8.DMEM_R2	21
**10**	screen_9CD14_R2	16231976	13161696	20355	113	0.08418	646.60752	90-8.DMEM_R2	29
**11**	screen_2DH18_R2	17647513	14811294	20355	113	0.08971	727.64893	26T.HPLM_R2	38
**12**	screen_2CH18_R2	15476734	13617594	20355	112	0.08669	669.00486	26T.HPLM_R2	38
**13**	screen_8DD12_R1	14179812	12368587	20355	97	0.08267	607.64367	ST-8814.DMEM_R1	24
**14**	screen_iDH7.7_R1	8586350	7504515	20355	94	0.105	368.68165	iHSC.HPLM_R1	32
**15**	screen_iCH7.7_R1	16664555	14428566	20355	92	0.1002	708.84628	iHSC.HPLM_R1	32
**16**	screen_9DD4.4_R2	8003532	5638235	20355	92	0.07781	276.99509	90-8.DMEM_R2	12
**17**	screen_iCD14_R1	14998694	10362323	20355	91	0.09358	509.07998	iHSC.DMEM_R1	29
**18**	screen_8DH8.7_R1	8485812	3874633	20355	88	0.08398	190.35289	ST-8814.HPLM_R1	37
**19**	screen_8CH3_R1	10948820	8681437	20355	87	0.07017	426.50145	ST-8814.HPLM_R1	21
**20**	screen_9DD14_R2	16210250	13326095	20355	84	0.0814	654.68411	90-8.DMEM_R2	29
**21**	screen_8CD2.1_R1	3256674	2488908	20355	83	0.08075	122.27502	ST-8814.DMEM_R1	8
**22**	screen_9CD4.4_R2	15362429	6424921	20355	83	0.08721	315.64338	90-8.DMEM_R2	12
**23**	screen_2DD9.3_R1	2030956	1546640	20355	80	0.08709	75.9833	26T.DMEM_R1	15
**24**	screen_9DD6.6_R2	4126924	3268581	20355	79	0.08475	160.57878	90-8.DMEM_R2	16
**25**	screen_iDH0_R1	11981907	9689994	20355	78	0.05766	476.04982	iHSC.HPLM_R1	4
**26**	screen_8CD4.5_R1	2869360	1687482	20355	76	0.09045	82.90258	ST-8814.DMEM_R1	12
**27**	screen_9CD2_R2	6410960	4063660	20355	75	0.0782	199.6394	90-8.DMEM_R2	8
**28**	screen_9DD2_R2	7464114	5806374	20355	75	0.0689	285.25542	90-8.DMEM_R2	8
**29**	screen_9DH26d_R1	17618040	12755286	20355	72	0.07775	626.64141	90-8.HPLM_R1	30
**30**	screen_8DD14_R1	19267141	15182586	20355	70	0.07573	745.88976	ST-8814.DMEM_R1	26
**31**	screen_iDD8.5_R1	9804945	8323850	20355	70	0.07608	408.93392	iHSC.DMEM_R1	18
**32**	screen_iDD14_R1	15271476	10603646	20355	68	0.0836	520.93569	iHSC.DMEM_R1	29
**33**	screen_9DD9.5_R2	14924609	12209122	20355	67	0.07051	599.80948	90-8.DMEM_R2	21
**34**	screen_8CH8.7_R1	6234090	5190192	20355	64	0.08152	254.98364	ST-8814.HPLM_R1	37
**35**	screen_iCD8.5_R1	6616196	5162947	20355	64	0.08533	253.64515	iHSC.DMEM_R1	18

**36**	screen_9DH0_R1	7891478	5967913	20355	64	0.06148	293.1915	90-8.HPLM_R1	4
**37**	screen_9CD6.6_R2	9390860	8358183	20355	63	0.07537	410.62063	90-8.DMEM_R2	16
**38**	screen_8DD2.1_R1	9992580	8518133	20355	62	0.05964	418.47865	ST-8814.DMEM_R1	8
**39**	screen_iDD5_R1	7784727	6643994	20355	62	0.06973	326.40599	iHSC.DMEM_R1	12
**40**	screen_8CD12_R1	16660002	12939483	20355	60	0.0774	635.69064	ST-8814.DMEM_R1	24
**41**	screen_8CH14_R1	18796730	10569221	20355	58	0.09927	519.24446	ST-8814.HPLM_R1	49
**42**	screen_iDD2.5_R1	8051268	6941373	20355	57	0.06407	341.01562	iHSC.DMEM_R1	8
**43**	screen_2DD0_R1	9798245	8091032	20355	56	0.05581	397.49605	26T.DMEM_R1	4
**44**	screen_2DD14_R1	15597801	12370265	20355	56	0.05947	607.72611	26T.DMEM_R1	20
**45**	screen_8DD4.5_R1	18910901	14367403	20355	56	0.06047	705.84146	ST-8814.DMEM_R1	12
**46**	screen_8DH0_R1	15696121	12939709	20355	56	0.05303	635.70174	ST-8814.HPLM_R1	4
**47**	screen_iDH4.7_R1	12005992	10289637	20355	56	0.07347	505.50906	iHSC.HPLM_R1	24
**48**	screen_8CD14_R1	17122156	13907042	20355	55	0.07863	683.22486	ST-8814.DMEM_R1	26
**49**	screen_9CH0_R1	6112404	4893344	20355	54	0.06391	240.4001	90-8.HPLM_R1	4
**50**	screen_iCD5_R1	2909260	2318736	20355	54	0.08788	113.91481	iHSC.DMEM_R1	12
**51**	screen_iCH0_R1	7840608	5700128	20355	54	0.06192	280.03577	iHSC.HPLM_R1	4
**52**	screen_8DH14_R1	17386109	12484376	20355	54	0.06955	613.33215	ST-8814.HPLM_R1	49
**53**	screen_2DD6.1_R1	6843156	5783900	20355	53	0.06413	284.15131	26T.DMEM_R1	11
**54**	screen_2DH16d_R2	20969871	13582981	20355	53	0.06076	667.3044	26T.HPLM_R2	20
**55**	screen_2DH0_R2	7810448	6711943	20355	50	0.05824	329.74419	26T.HPLM_R2	4
**56**	screen_2CH16d_R2	10600172	8738443	20355	49	0.07524	429.30204	26T.HPLM_R2	20
**57**	screen_iDD0_R1	17727880	12714867	20355	49	0.05373	624.65571	iHSC.DMEM_R1	4
**58**	screen_2CD9.3_R1	8836421	4288009	20355	48	0.07256	210.66121	26T.DMEM_R1	15
**59**	screen_8DH21d_R1	14615659	8594497	20355	48	0.06342	422.23026	ST-8814.HPLM_R1	25
**60**	screen_8CH21d_R1	7585294	6045532	20355	47	0.07034	297.00477	ST-8814.HPLM_R1	25
**61**	screen_9DD0_R2	18886352	15432169	20355	46	0.04967	758.15127	90-8.DMEM_R2	4
**62**	screen_2CD14_R1	15713366	12770497	20355	45	0.05999	627.3887	26T.DMEM_R1	20
**63**	screen_8DD0_R1	18556476	15093569	20355	45	0.04961	741.51653	ST-8814.DMEM_R1	4
**64**	pDNA_HD_amplified	4620982	3969959	20355	45	0.05969	195.03606	pDNA	0
**65**	screen_iCD2.5_R1	9149893	7023685	20355	44	0.06621	345.05944	iHSC.DMEM_R1	8
**66**	screen_2CD6.1_R1	4794172	3941495	20355	43	0.06978	193.63768	26T.DMEM_R1	11
**67**	screen_iCD0_R1	14213821	10116583	20355	43	0.05685	497.00727	iHSC.DMEM_R1	4
**68**	screen_iCH4.7_R1	11916302	9828092	20355	42	0.07456	482.83429	iHSC.HPLM_R1	24
**69**	screen_2CD0_R1	10838796	9069441	20355	35	0.05456	445.5633	26T.DMEM_R1	4
**70**	screen_8CH0_R1	17565354	12751166	20355	34	0.05253	626.43901	ST-8814.HPLM_R1	4

**71**	screen_9CD0_R2	16432500	13437502	20355	31	0.0517	660.15731	90-8.DMEM_R2	4
**72**	screen_2CH0_R2	13573183	10776325	20355	29	0.05402	529.41906	26T.HPLM_R2	4
**73**	screen_8CD0_R1	20267766	15323545	20355	28	0.0498	752.81479	ST-8814.DMEM_R1	4
**74**	pDNA_HC_amplified	6452324	5420959	20355	23	0.05571	266.32076	pDNA	0
**75**	T550	23292260	14531368	49349	19	0.04157	294.474214	pDNA	0
**76**	T0_HOS	54356837	31602045	49349	416	0.1445	640.37863	HOS	4
**77**	T1_HOS	42223797	18944596	49349	241	0.1346	383.89017	HOS	6
**78**	T2_HOS	56696453	35500969	49349	69	0.08694	719.385783	HOS	8
**79**	T3_HOS	49670643	29031031	49349	158	0.1153	588.280026	HOS	10
**80**	T4_HOS	48762401	26890026	49349	148	0.1099	544.895054	HOS	12
**81**	T5_HOS	49911551	27755288	49349	160	0.107	562.42858	HOS	14
**82**	T6_HOS	52807602	29496893	49349	454	0.1356	597.720177	HOS	18
**83**	T7_HOS	31855989	11814989	49349	1258	0.1824	239.416989	HOS	21
**84**	T0_OHS	45055715	29355014	49349	45	0.07473	594.845164	OHS	5
**85**	T1_OHS	41016675	24646258	49349	88	0.09376	499.427709	OHS	9
**86**	T2_OHS	41049983	24406352	49349	104	0.1008	494.566293	OHS	13
**87**	T3_OHS	38308768	22994730	49349	89	0.0972	465.961418	OHS	15
**88**	T4_OHS	36047101	22234709	49349	100	0.1001	450.560477	OHS	17
**89**	T5_OHS	38233539	21214865	49349	404	0.1478	429.894527	OHS	21
**90**	T6_OHS	29265166	15098153	49349	184	0.1302	305.946483	OHS	25
**91**	T7_OHS	41589669	25715000	49349	481	0.1576	521.08452	OHS	29

Supplementary Table 9. Effective cell kill concentrations per cell line

Cell Line	Puromycin (µg/mL)
STS26T	1.7
90-08	1.7
ST-8814	3
iHSC	1.7
HOS	0.5
OHS	1

Supplementary Table 10. Next-Generation-Sequencing primers.

Name	Type	Sequence
P7_KERMIT_1	i7	CAAGCAGAAGACGGCATACGAGATTCGCCTTAGTGACTGGAGTTCAGACGTGTGCTCTTCCGATCTTCTACTATTCTTTCCCCTGCACTGT
P7_KERMIT_2	i7	CAAGCAGAAGACGGCATACGAGATCTAGTACGGTGACTGGAGTTCAGACGTGTGCTCTTCCGATCTTCTACTATTCTTTCCCCTGCACTGT
P7_KERMIT_3	i7	CAAGCAGAAGACGGCATACGAGATGCTCAGGAGTGACTGGAGTTCAGACGTGTGCTCTTCCGATCTTCTACTATTCTTTCCCCTGCACTGT
P7_KERMIT_4	i7	CAAGCAGAAGACGGCATACGAGATAGGAGTCCGTGACTGGAGTTCAGACGTGTGCTCTTCCGATCTTCTACTATTCTTTCCCCTGCACTGT
P7_KERMIT_5	i7	CAAGCAGAAGACGGCATACGAGATTTCTGCCTGTGACTGGAGTTCAGACGTGTGCTCTTCCGATCTTCTACTATTCTTTCCCCTGCACTGT
P7_KERMIT_6	i7	CAAGCAGAAGACGGCATACGAGATCATGCCTAGTGACTGGAGTTCAGACGTGTGCTCTTCCGATCTTCTACTATTCTTTCCCCTGCACTGT
P7_KERMIT_7	i7	CAAGCAGAAGACGGCATACGAGATGTAGAGAGGTGACTGGAGTTCAGACGTGTGCTCTTCCGATCTTCTACTATTCTTTCCCCTGCACTGT
P7_KERMIT_8	i7	CAAGCAGAAGACGGCATACGAGATCCTCTCTGGTGACTGGAGTTCAGACGTGTGCTCTTCCGATCTTCTACTATTCTTTCCCCTGCACTGT
P5_ARGON_9	i5	AATGATACGGCGACCACCGAGATCTACACTATAGCCTACACTCTTTCCCTACACGACGCTCTTCCGATCTTTGTGGAAAGGACGAAACACCG
P5_ARGON_10	i5	AATGATACGGCGACCACCGAGATCTACACATAGAGGCACACTCTTTCCCTACACGACGCTCTTCCGATCTTTGTGGAAAGGACGAAACACCG
P5_ARGON_11	i5	AATGATACGGCGACCACCGAGATCTACACCCTATCCTACACTCTTTCCCTACACGACGCTCTTCCGATCTTTGTGGAAAGGACGAAACACCG
P5_ARGON_12	i5	AATGATACGGCGACCACCGAGATCTACACGGCTCTGAACACTCTTTCCCTACACGACGCTCTTCCGATCTTTGTGGAAAGGACGAAACACCG
P5_ARGON_13	i5	AATGATACGGCGACCACCGAGATCTACACAGGCGAAGACACTCTTTCCCTACACGACGCTCTTCCGATCTTTGTGGAAAGGACGAAACACCG
P5_ARGON_14	i5	AATGATACGGCGACCACCGAGATCTACACTAATCTTAACACTCTTTCCCTACACGACGCTCTTCCGATCTTTGTGGAAAGGACGAAACACCG
P5_ARGON_15	i5	AATGATACGGCGACCACCGAGATCTACACCAGGACGTACACTCTTTCCCTACACGACGCTCTTCCGATCTTTGTGGAAAGGACGAAACACCG
P5_ARGON_16	i5	AATGATACGGCGACCACCGAGATCTACACGTACTGACACACTCTTTCCCTACACGACGCTCTTCCGATCTTTGTGGAAAGGACGAAACACCG

Supplementary Table 11. Programme gRNA cassette region PCR amplification.

Stage	Temperature (°C)	Time	Number of Cycles
Initial Denaturation	98	03:00	1x
Denaturation	98	00:15	5x (Stage B)
Annealing	65	00:30	5x (Stage B)
Extension	72	00:30	5x (Stage B)
Denaturation	98	00:15	8x (Stage C)
Annealing	63	00:30	8x (Stage C)
Extension	72	00:30	8x (Stage C)
Denaturation	98	00:15	12x (Stage D)
Annealing	61	00:30	12x (Stage D)
Extension	72	00:30	12x (Stage D)
Final Extension	72	07:00	1x
Hold	4	Infinity	1x

Supplementary Table 12. External datasets used for this study.

Dataset	Source
Common Essential Genes	[25]
DepMap gRNA Count Matrix	Data | DepMap Portal. https://depmap.org/portal/data_page/?tab=allData.
Common non-essential Genes	Chronos: Gene Fitness Effect Inference from CRISPR. https://figshare.com/articles/dataset/Chronos_Gene_Fitness_Effect_Inference_from_CRISPR/14067047.
Screen Gene Effect scores	Data | DepMap Portal. https://depmap.org/portal/data_page/?tab=allData.
Compact and non-compact CRISPRi screen data	https://link.springer.com/article/10.1186/s13059-023-03132-3#availability-of-data-and-materials
